# miR-338-3p Is Down-Regulated by Hepatitis B Virus X and Inhibits Cell Proliferation by Targeting the 3′-UTR Region of CyclinD1

**DOI:** 10.3390/ijms13078514

**Published:** 2012-07-09

**Authors:** Xiaoyu Fu, Deming Tan, Zhouhua Hou, Zhiliang Hu, Guozhen Liu

**Affiliations:** 1 Department of Infectious Disease, Xiangya Hospital, Central South University, Changsha, Hunan 410008, China; E-Mails: goodwish0321@yahoo.com.cn (X.F.); houzhouhua@yahoo.com.cn (Z.H.); guozhenliu.2008@yahoo.com.cn (G.L.); 2 The Second Hospital of Nanjing, Nanjing, Jiangsu, 210000, China; E-Mail: huzhiliang87@yahoo.com.cn

**Keywords:** hepatocellular carcinoma, hepatitis B virus X protein, miR-338-3p, CyclinD1, cell proliferation

## Abstract

Hepatitis B virus X protein (HBx) is recognized as an oncogene in hepatocellular carcinoma (HCC). HBx regulates microRNA expression, including down-regulating miR-338-3p in LO2 cells. Here, we investigated miR-338-3p function in HBx-mediated hepatocarcinogenesis. In 23 HBV-infected HCC clinical patient tumor and adjacent non-tumor control tissues, 17 and 19 tumors expressed HBx mRNA and protein, respectively. When considered as a group, HBV-infected HCC tumors had lower miR-338-3p expression than controls; however, miR-338-3p was only significantly down-regulated in HBx-positive tumors, indicating that HBx inversely correlated with miR-338-3p. Functional characterization of miR-338-3p indicated that miR-338-3p mimics inhibited cell proliferation by inducing cell cycle arrest at the G1/S phase as assessed by EdU and cell cycle assays in HBx-expressing LO2 cells. CyclinD1, containing two putative miR-338-3p targets, was confirmed as a direct target using 3′-UTR luciferase reporter assays from cells transfected with mutated binding sites. Mutating the 2397–2403 nt binding site conferred the greatest resistance to miR-338-3p suppression of CyclinD1, indicating that miR-338-3p suppresses CyclinD1 at this site. Overall, this study demonstrates that miR-338-3p inhibits proliferation by regulating CyclinD1, and HBx down-regulates miR-338-3p in HCC. This newly identified miR-338-3p/CyclinD1 interaction provides novel insights into HBx-mediated hepatocarcinogenesis and may facilitate therapeutic development against HCC.

## 1. Introduction

Hepatocellular carcinoma (HCC) is known as one of the most common malignant tumors worldwide, and accounts for nearly 700,000 deaths per year [1]. Overall, more than 50% of HCC cases are attributed to chronic and persistent hepatitis B virus (HBV) infections [2]. HBV has been suggested to contribute to oncogenesis through two major mechanisms, acting alone or together: (1) viral genomic fragments integrating into the human genome that then activate oncogenes or inhibit tumor suppressor genes, and (2) viral products inducing cellular pathways that lead to carcinogenesis [3]. Further studies revealed that, of the four HBV genes, HBV X (HBx) is the most common viral open reading frame that integrates into the host genome. The HBx protein is of significant interest because it plays an important role in hepatocarcinogenesis. It modulates cell sensitivity to cytokines and growth factors, trans-activates multiple cellular and viral genes [4], and forms complexes with various host cell proteins such as the p53 tumor suppressor protein [5]. However, despite extensive studies, the mechanism of how HBx promotes malignant cell transformation remains unclear [6].

MicroRNAs (miRNAs) are a class of small (~22 nucleotide) RNAs that are evolutionarily conserved among diverse organisms and are responsible for regulating the post-transcriptional expression of their target genes. Multiple studies have indicated that miRNA expression changes are associated with carcinogenesis and the progression of various cancers [7,8]. HCC in particular is a complex disease involving epigenetic instability, chromosomal instability, and the abnormal expression of both coding and noncoding genes, including miRNAs [9]. Large-scale miRNA expression profile analyses of HCC samples revealed that miRNA deregulation is a frequent event in HCC and is closely related to HCC development [10,11]. Because the HBx protein trans-activates various cellular genes and pathways, we wondered whether HBx might regulate miRNA expression. This was first suggested in a 2010 study showing that the HBx protein down-regulated let-7a and promoted cell proliferation by targeting signal transducer and activator of transcription 3 (STAT 3) [12].

Our unpublished miRNA microarray and real-time polymerase chain reaction (PCR) studies indicated that miR-338-3p expression was down-regulated in HBx-expressing LO2 cells compared with control LO2/pcDNA cells. Furthermore, HBx-expressing LO2 cells overexpressed CyclinD1 [13], which has been defined as a potential target of miR-338-3p *in silico*. In this study, we investigated the function of miR-338-3p in HBx-expressing LO2 cells, showing that miR-338-3p was down-regulated and regulated CyclinD1 in HBV-related HCC. Our results suggest that miR-338-3p may represent a novel factor through which the HBx protein can act to modulate cell proliferation and promote hepatocarcinogenesis.

## 2. Results

### 2.1. miR-338-3p Is Significantly Reduced in HBx-Positive Tumor Tissues and Inversely Correlates with HBx Expression

**Figure 1:**
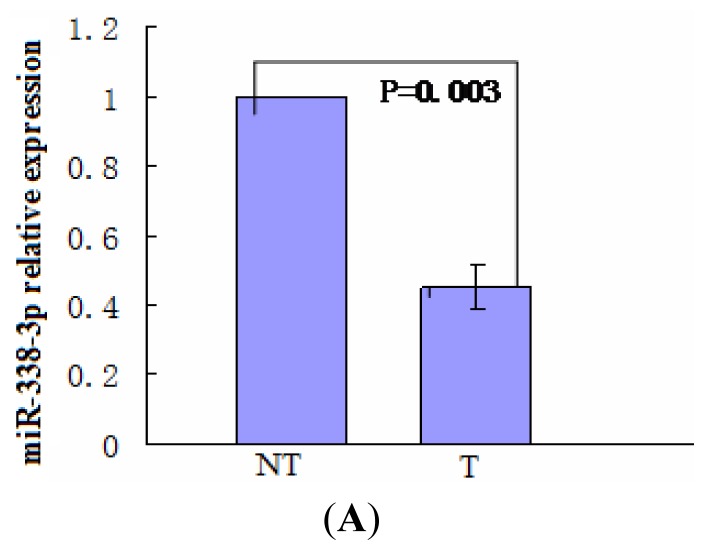

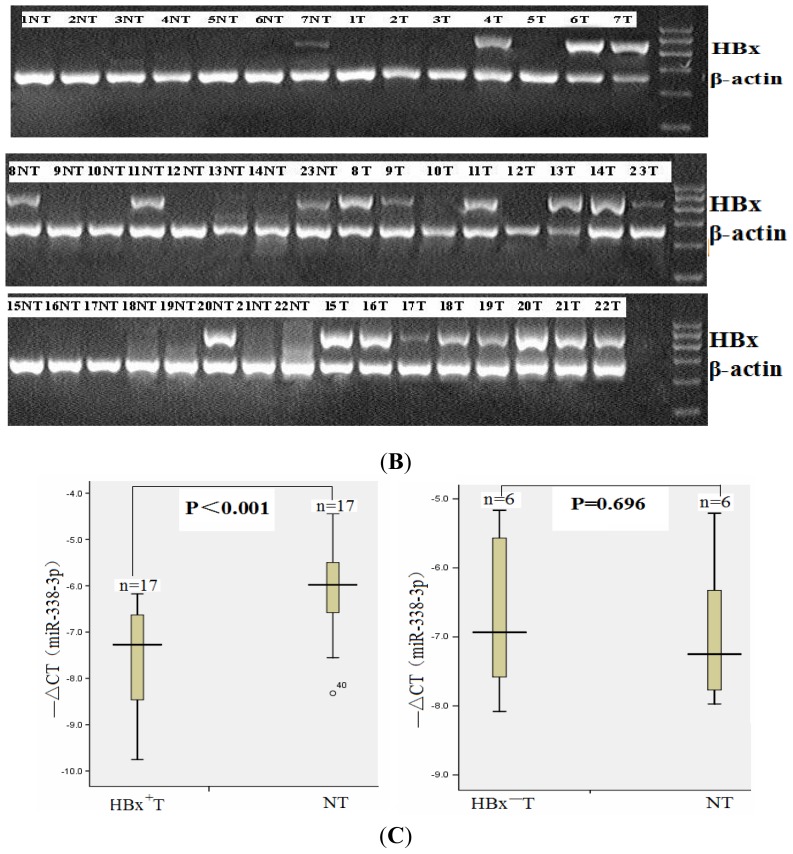
miR-338-3p and Hepatitis B virus X protein (HBx) expression in 23 paired tumor (T) and control non-tumor (NT) tissues from hepatocellular carcinoma (HCC) patients measured by RT-PCR. (**A**) qRT-PCR analysis of miR-338-3p expression levels in the 23 paired tissue samples normalized to U6 using the 2^−ΔΔCT^ method. (**B**) HBx expression in 23 paired tissue samples. HBx mRNA was detected in 17 tumors cases (17/23, 74%). Among those 17, 5 adjacent non-tumors were also HBx positive. (**C**) Only in HBx-positive HCC tumors, miR-338-3p expression was significantly reduced compared to the paired adjacent non-tumor tissues. Box plots represent respective miR-338-3p ΔCT values normalized to U6 in the 23 paired tissue samples. Left panel: miR-338-3p expression in 17 HBx-positive tumors in HCC patients compared to the adjacent non-tumor tissues (*p* < 0.001). Right panel: miR-338-3p expression in 6 HBx-negative tumors in HCC patients compared to the adjacent non-tumor tissues respectively (*p* = 0.696).

To evaluate the clinical relevance of HBx and miR-338-3p expression, miR-338-3p and HBx mRNA were examined in 23 HCC tumor tissues paired with control surrounding normal tissue by qRT-PCR (using the 2^−ΔΔCT^ method) in 3 independent experiments. miR-338-3p expression was reduced in HCC tumor tissues compared to the adjacent non-tumor tissues (Figure 1A). Using RT-PCR, HBx mRNA expression was detected in 74% (17/23) of the tumor tissues; within these 17 cases, only 5 of the corresponding control adjacent non-tumor tissues were HBx positive (Figure 1B). This result indicated that HBx mRNA closely correlated with HCC tumorigenesis. As we previously observed that HBx expression in an LO2 cell line correlated with lower miR-338-3p expression, we tested whether this association could also be observed in patient tumor tissue. Clinicopathological characteristics and miR-338-3p expression of 23 patients with HCC is shown in Table S1. Indeed, we found that miR-338-3p expression was significantly down-regulated only in HBx-positive tumor tissues when compared to adjacent non-tumor tissues with low or no HBx mRNA expression (*p* < 0.001). In contrast, miR-338-3p expression in HBx-negative tumor tissues was not significantly different from the non-tumor tissues, although expression was slightly higher in the tumor tissues (Figure 1C, *p* > 0.05).

**Figure 2:**
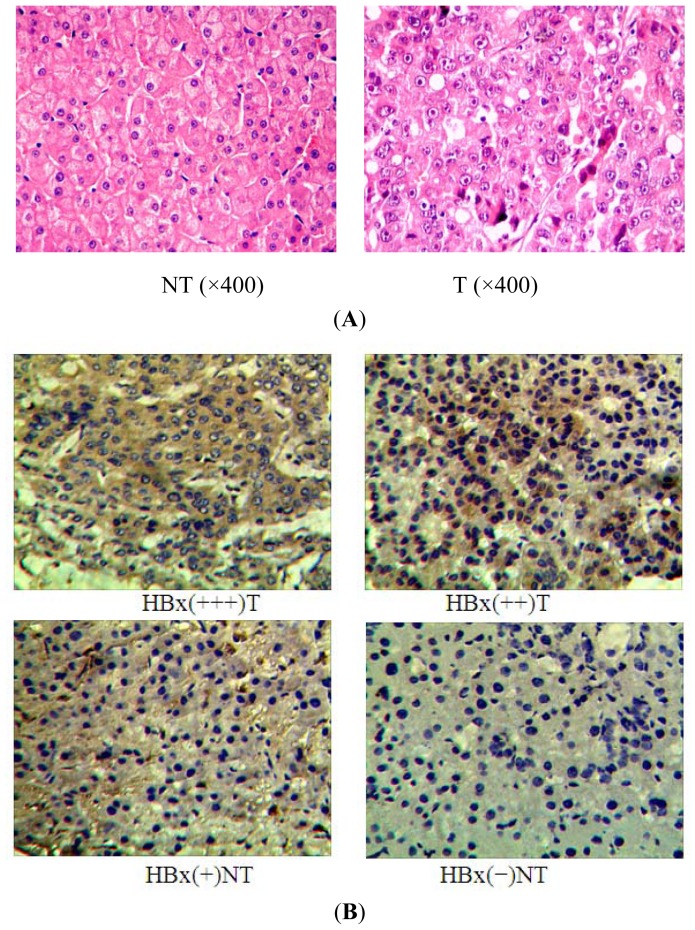

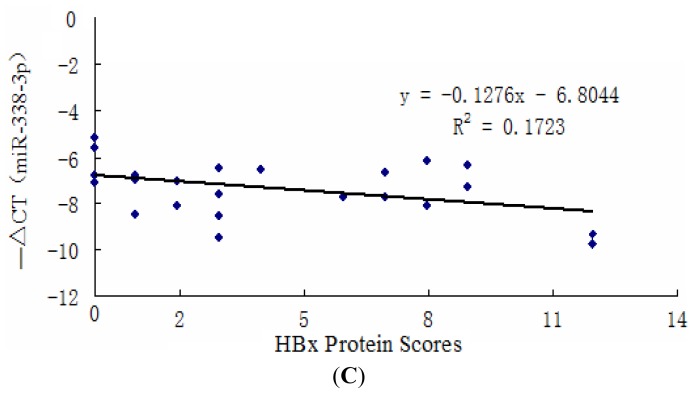
HBx protein level expression in 23 paired tumor (T) and control non-tumor (NT) tissues from HCC patients measured by immunohistochemistry. (**A**) Morphologic changes were analyzed in the 23 paired paraffin-embedded liver tissue samples by H & E staining, and representative images from tumor or control non-tumor tissue is shown. (**B**) HBx protein levels in tumor or adjacent control non-tumor tissue from HCC patients were evaluated by immunohistochemical staining. Images that represent each level of the semi-quantitative scoring system are shown: negative (−); weakly positive (+); moderately positive (++); and strongly positive (+++). 19 tumor tissues exhibited higher HBx expression (19/23, 82.6%). Among these 19, 5 adjacent non-tumor tissues exhibited low HBx expression. (**C**) Inverse correlation between HBx protein expression and miR-338-3p expression in the 23 HCC tissues. miR-338-3p expression in these samples was measured by qRT-PCR (as shown above). Correlation between the ΔCT values and HBx protein expression scores is statistically significant by Pearson’s correlation analysis (*p* = 0.003, *r* = −0.456).

Moreover, we analyzed HBx protein expression in the 23 tumor and control non-tumor tissue pairs by immunohistochemistry and subsequent semi-quantitative analysis using a scoring system based on the percentage of HBx positive cells as well as the staining intensity color of the positive cells. The identities of both the tumor and noncancerous samples were first confirmed histologically by H&E staining (Figure 2A). The data showed that 82.6% (19/23, where only 17 of these were positive for HBx mRNA) of the tumor tissues exhibited higher HBx protein staining scores (Figure 2B). Although the data was highly variable due to the inherent variability in clinical samples from human tissue, a statistically significant negative correlation existed between miR-338-3p mRNA and HBx protein expression in the 19 paired HBx-positive tumor and control non-tumor tissues (Figure 2C, *p* = 0.003).

### 2.2. miR-338-3p Inhibits Cell Proliferation

**Figure 3:**
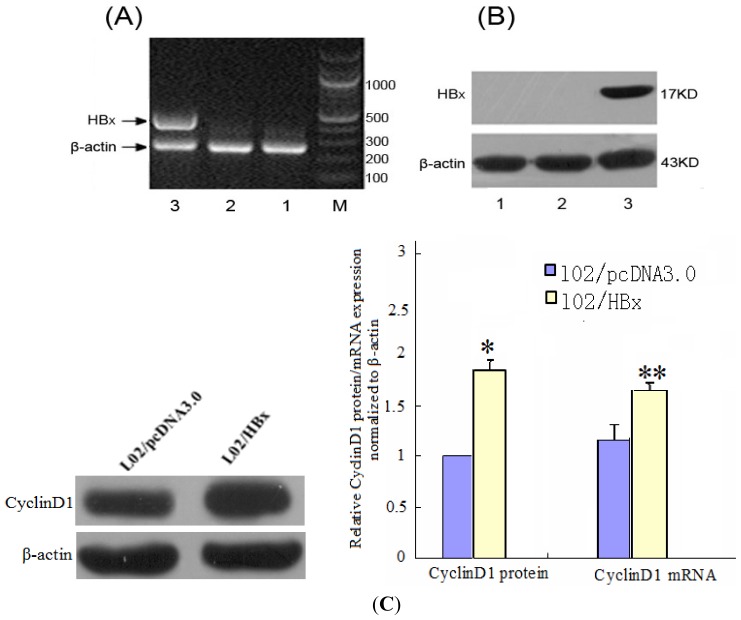
The identification of stable HBx transfection in LO2 cells and the CyclinD1 expression in transfected cells. (**A**) HBx gene expression in LO2 cells was identified by RT-PCR. (**B**) Western blot showing the expression of HBx in LO2 cells. 1: LO2; 2: LO2/pcDNA3.0; 3: LO2/HBx; M: marker. (**C**) CyclinD1 mRNA and protein level in LO2/HBx cells were measured by Western blot and RT-PCR using β-actin as a control, and then compared with CyclinD1 expression in control LO2/pcDNA3.0 cells. (* *p* = 0.017, ** *p* = 0.029).

To further investigate the functional consequences of the correlation we observed between miR-338-3p and HBx, we developed an *in vitro* cell model to specifically test the role of HBx expression on miR-338-3p function. We established a cell line expressing wild-type HBx by transfecting a non-tumorigenic human hepatocyte cell line, LO2, with an HBx-containing plasmid or a control empty plasmid. RT-PCR analysis confirmed HBx gene expression, indicating that the HBx gene was successfully introduced into the host genome of the engineered LO2 cells (Figure 3A), and western blotting confirmed HBx protein expression (Figure 3B). Our data show that stably HBx-transfected LO2 cells were successfully established. As the cyclin proteins are known to regulate cell proliferation and cell cycle, we tested whether CyclinD1 was differentially expressed in the engineered LO2 cells. PCR and western blot results showed that CyclinD1 mRNA and protein levels, respectively, were up-regulated in cells transfected with HBx, when compared with those transfected with pcDNA3.0 (Figure 3C). This result indicated that HBx increased CyclinD1 expression in hepatocytes.

**Figure 4:**
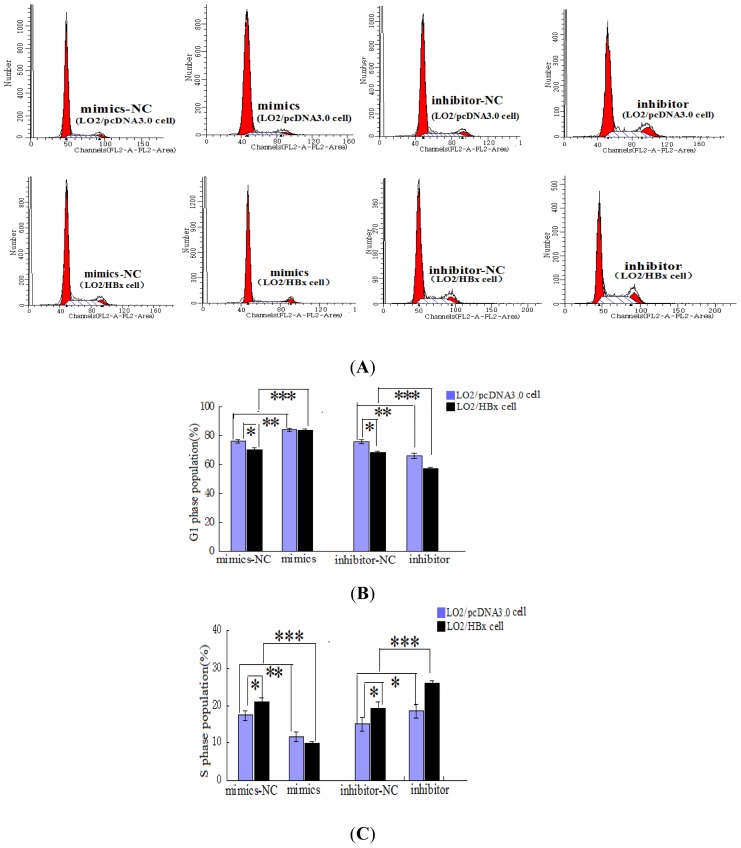
Effect of miR-338-3p expression on cell cycle. LO2/HBx cells or control LO2/pcDNA3.0 cells were transfected with miR-338-3p mimics, miR-338-3p inhibitor, or their respective control RNA and grown for 48 h before flow cytometry analysis. (**A**) Representative cell cycle profiles for LO2/HBx cells or control LO2/pcDNA3.0 cells treated with miR-338-3p mimics or inhibitor compared to their representative control RNA or mock-transfected cells. (**B,C**) Analysis of the G1 (B) or S (C) phase population from (A). Data shows that miR-338-3p mimics induced cell cycle arrest while inhibition of miR-338-3p inhibition encouraged increased cell growth. Values in (B) and (C) are shown as means ± SD from 3 separate experiments. (* *p <* 0.05, ** *p <* 0.01, *** *p <* 0.001).

**Figure 5:**
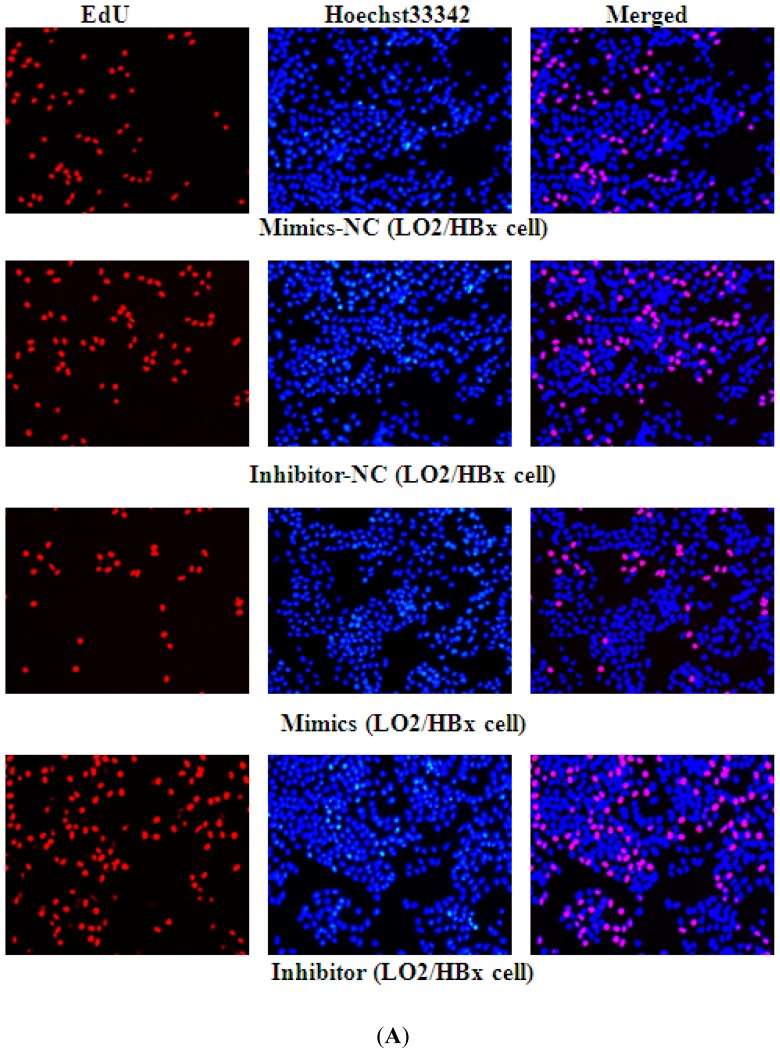

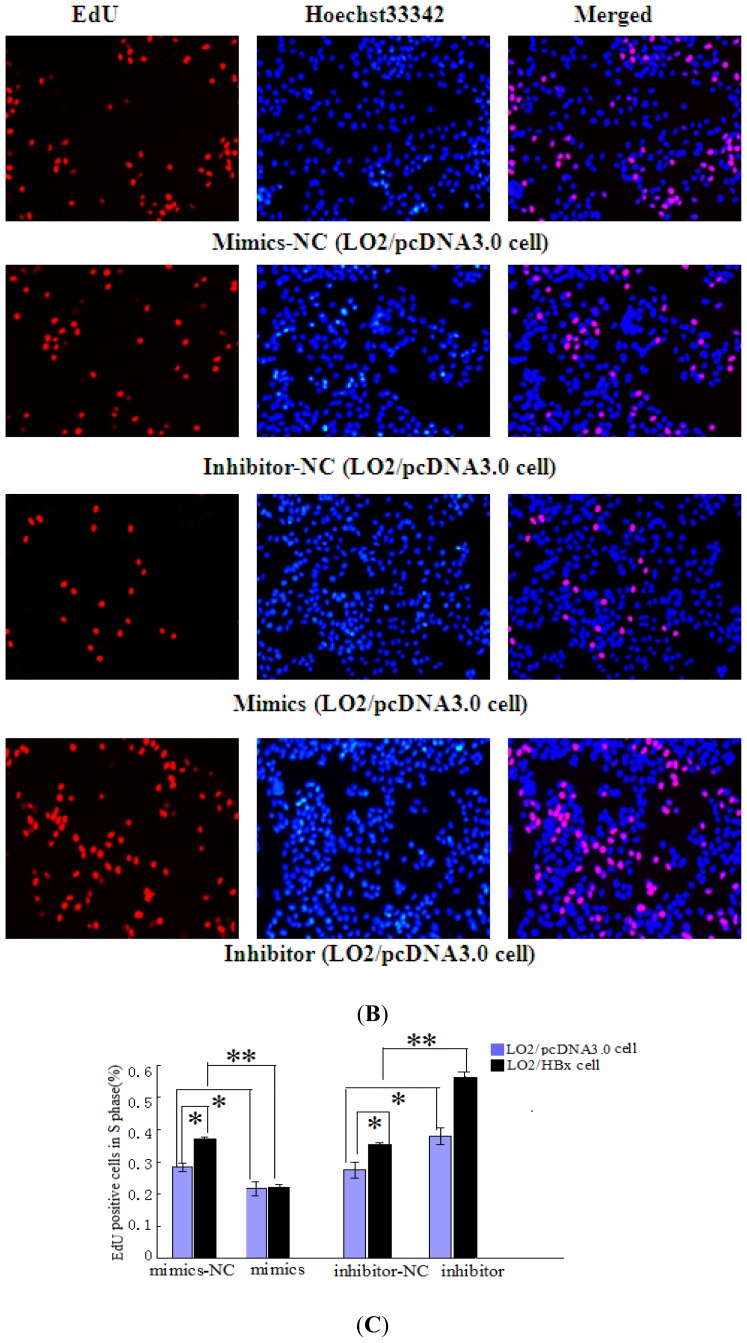
miR-338-3p overexpression inhibits cellular DNA replication in LO2/HBx cells, whereas miR-338-3p inhibition demonstrates increased cell growth by EdU cell proliferation analysis. (**A**,**B**) Representative immunofluorescent images of cell proliferation measured by EdU staining after miR-338-3p mimics or inhibitor transfection compared to their negative control transfection in LO2/HBx and LO2/pcDNA3.0 control cells (100× magnification). (**C**) Rate of EdU-positive cells in S phase. (* *p* < 0.05, ** *p* < 0.01).

To investigate the functional role of the HBx-associated miR-338-3p down-regulation, we used cell cycle analysis by FACS and EdU assays to evaluate the effect of miR-338-3p on cell proliferation. We first evaluated the effect of HBx on proliferation and found that LO2/HBx cells exhibited a significantly lower cell percentage in G1 phase and a significantly higher cell percentage in S phase (Figure 4A–C, *p* < 0.05), indicating increased cell proliferation. The effect of miR-338-3p specifically was then tested. While cells transfected with negative control RNAs progressed through different phases of the cell cycle, cells transfected with miR-338-3p mimics displayed a significantly higher frequency of cells in the G1 phase and a lower frequency of cells in S phase (Figure 4A–C, *p* < 0.001). In HBx-expressing cells, miR-338-3p mimic expression was able to restore the lower proliferative capacity exhibited by normal non-HBx expressing cells. In contrast, cells transfected with the miR-338-3p inhibitor showed a significant decrease in the proportion of cells in the G1 phase and an increase in the proportion of cells in S phase (Figure 4A–C, Table S2, *p* < 0.001). These data indicated that miR-338-3p functions to inhibit cell proliferation. Next, we used the EdU incorporation assay, a more sensitive and specific method [14], to evaluate miR-338-3p function. We found that the number of EdU-positive cells among the cells transfected with the miR-338-3p mimics was significantly reduced, while the number of EdU cells was significantly increased in the miR-338-3p inhibitor group compared with the cells transfected with negative control RNAs (Figure 5A,B). Confirming the cell cycle analysis data, these data indicated that miR-338-3p inhibited cell proliferation in HBx-expressing LO2 cells.

### 2.3. CyclinD1 Is a Direct Target of miR-338-3p, and Position 2397–2403 nt Is the Major Target Site

**Figure 6:**
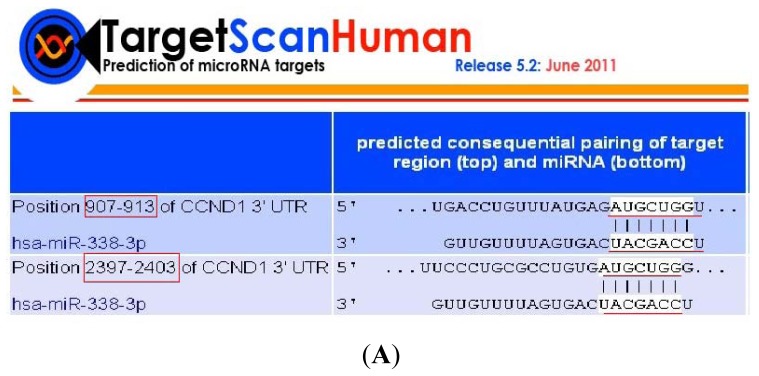

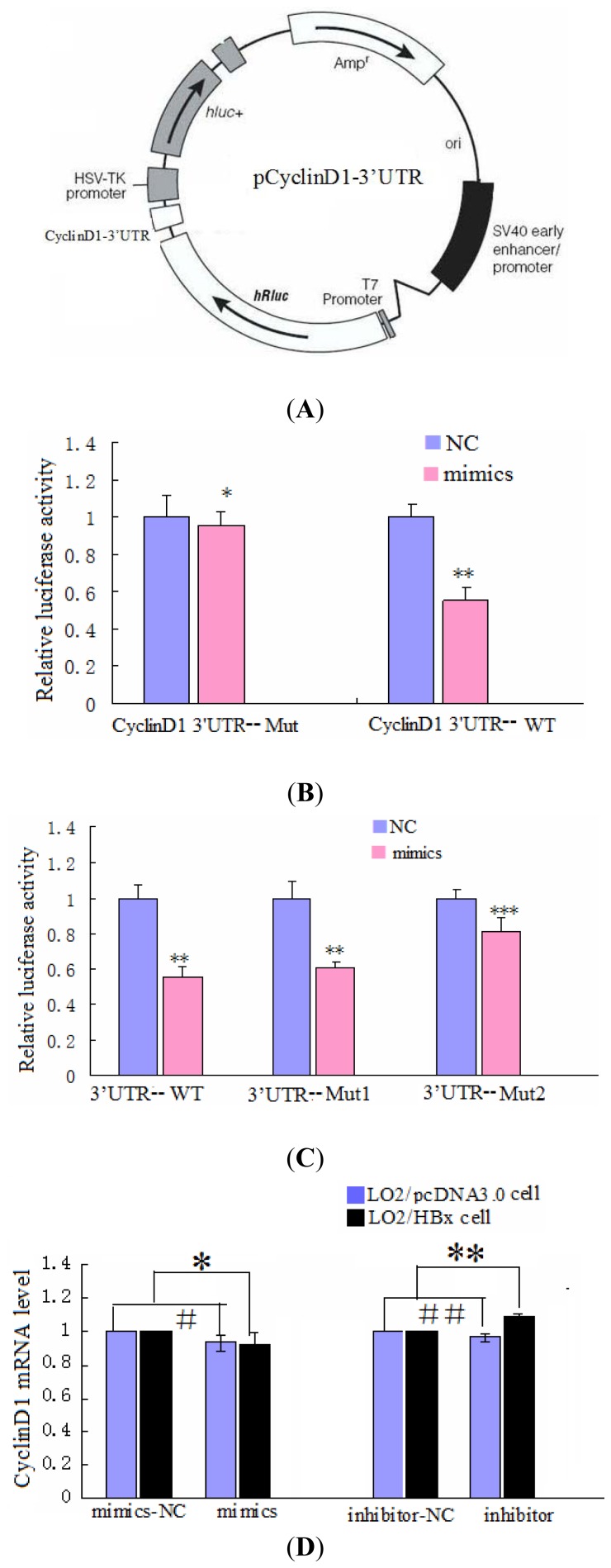

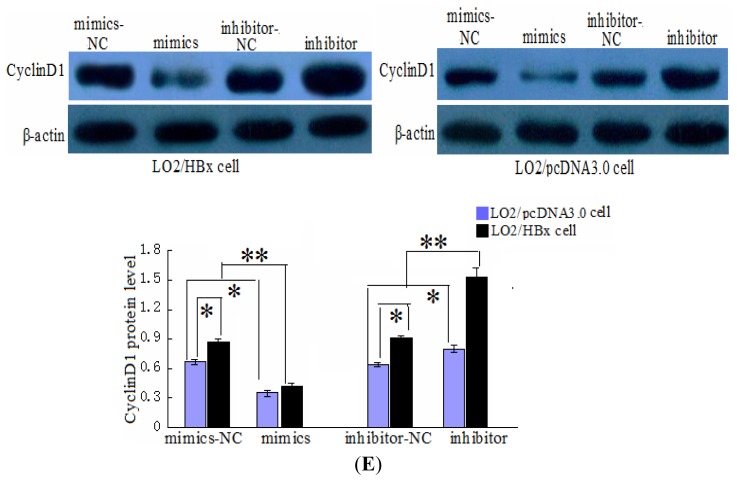
miR-338-3p targets CyclinD1. (**A**) Schematic diagram of the putative interaction sites and the constructs used to validate that miR-338-3p targets the 3′-UTR of CyclinD1. (**B**) Dual luciferase assay of LO2/HBx cells co-transfected with Renilla luciferase plasmids containing CyclinD1-WT or -Mut 3′-UTR and miR-338-3p mimics or negative control RNA (* *p* = 0.404, ** *p* < 0.001). (**C**) 2397–2403 nt in the 3′-UTR of CyclinD1 is the major functional target site of miR-338-3p. The effect of miR-338-3p and negative control RNA on CyclinD1 3′-UTR Mut1 and Mut2 reporter constructs in LO2/HBx cells were determined 48 h after transfection. Renilla luciferase values normalized to Firefly luciferase activity are presented (* *p <* 0.001, *** *p* = 0.04). (**D**) qRT-PCR was used to evaluate the CyclinD1 mRNA expression in LO2/HBx cells transfected with miR-338-3p mimics, inhibitor, or their respective negative control; CyclinD1 mRNA was normalized to GAPDH and data was analyzed using the 2^−ΔΔCT^ method (* *p* = 0.218, ** *p* = 0.1, ^#^ *p* = 0.254, ^##^ *p* = 0.417). (**E**) CyclinD1 protein expression after miR-338-3p mimics, inhibitor, or negative control transfection (* *p* < 0.01, ** *p* < 0.001). Data are shown as mean ± SD from at least three independent experiments.

As predicted by several *in silico* analyses, including TargetScan [15], CyclinD1 was identified as one of the candidate genes potentially regulated by miR-338-3p. As shown in Figure 6A, the CyclinD1-encoded mRNA contains two 3′-UTR elements that are partially complementary to miR-338-3p, indicating that miR-338-3p could directly target either of the two sites. To validate whether miR-338-3p directly regulates CyclinD1, the CyclinD1-complementary sites were cloned into the 3′-UTR of the Renilla luciferase gene with or without mutations. This construct was co-transfected into LO2/HBx cells along with miR-338-3p mimics or negative control RNA, and the level of luciferase enzyme activity was measured to determine the effects of miR-338-3p on luciferase translation. miR-338-3p overexpression suppressed the luciferase activity of the WT CyclinD1 3′-UTR construct with respect to the negative control, whereas the mutation of both putative miR-338-3p-binding sites in the CyclinD1 3′-UTR construct abolished this suppression, confirming the specificity of these CyclinD1 target sites for miR-338-3p suppressive activity (Figure 6B).

To determine which putative target site in the CyclinD1 3′-UTR is most critical for miR-338-3p suppression, each site was mutated and cloned separately. As shown in Figure 6C, introduction of the miR-338-3p mimic induced significantly lower luciferase activity in LO2/HBx cells transfected with either the 3′-UTR WT or the 3′-UTR Mut1 reporter construct (*p* < 0.001) as well as the as 3′-UTR Mut2 (*p* = 0.04) compared to the negative control RNA. However, the cells containing the 3′-UTR Mut2 construct demonstrated significantly more luciferase activity than the 3′-UTR WT or the 3′-UTR Mut1 constructs in the presence of miR-338-3p, indicating that this mutated construct of the second target site conferred resistance to the miR-338-3p–mediated suppression of CyclinD1. These results indicate that the 2397–2403 nt sequence of the CyclinD1 3′-UTR is the major target of miR-338-3p.

To directly test the validity of the putative target by evaluating CyclinD1 protein expression after miR-338-3p, HBx-expressing LO2 cells were transfected with miR-338-3p mimics or the miR-338-3p inhibitor as well as their respective negative controls. We first assayed for mRNA expression and found no change in CyclinD1 mRNA among any of the groups by qRT-PCR (Figure 6D). CyclinD1 protein was affected, however, because miR-338-3p overexpression down-regulated the endogenous CyclinD1 protein relative to the control RNA by western blot analysis, while the inhibition of miR-338-3p up-regulated the CyclinD1 protein (Figure 6E). This data indicated that CyclinD1 protein expression is directly regulated by miR-338-3p.

**Figure 7:**
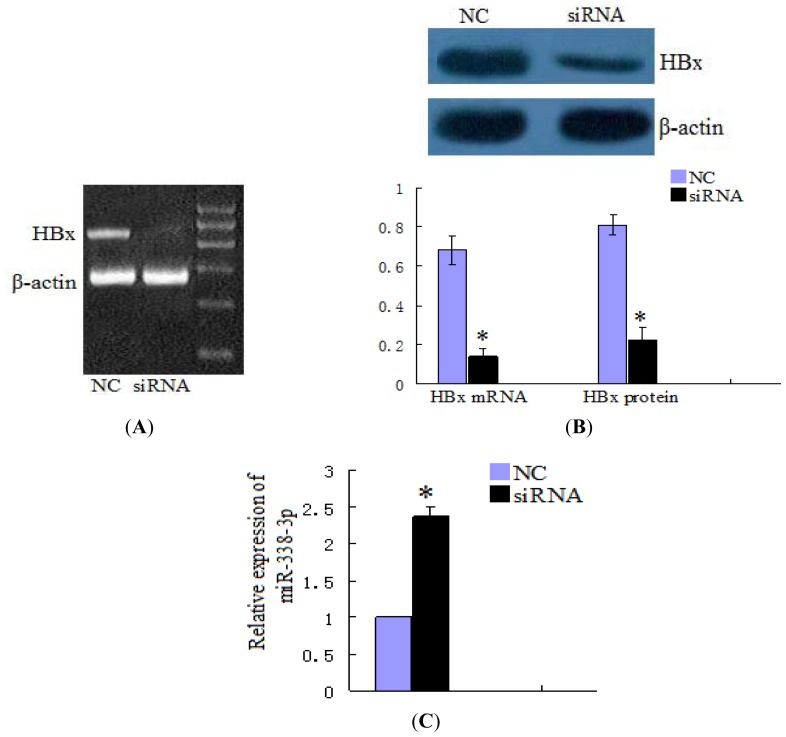

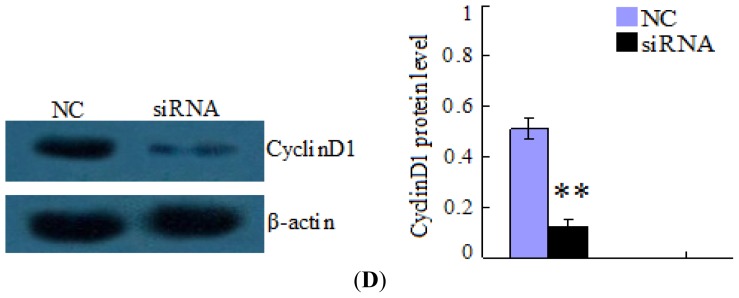
miR-338-3p and CyclinD1 expression after knocking-down HBx in HBx-expressing cells. (**A**,**B**) HBx mRNA and protein expression levels after HBx siRNA transfection compared with control RNA in LO2/HBx cells (* *p* < 0.001). (**C**) Relative expression by qRT-PCR analysis of miR-338-8p using the 2^−ΔΔCT^ method after HBx changes in LO2/HBx cells (* *p* = 0.013). (**D**) CyclinD1 levels after the transfection of the HBx siRNA or negative control in HBx-expressing cells (** *p* < 0.001). Data are shown as means ± SD from 3 separate experiments.

Further confirming that HBx expression upstream of miR-338-3p could regulate CyclinD1 expression, we found that a high level of HBx expression in LO2/HBx cells correlated with increased CyclinD1 expression by PCR and western blot analysis. Microarray and real-time RT-PCR analysis revealed that miR-338-3p was down-regulated in LO2/HBx cells compared with the control LO2/pcDNA3.0 cells, indicating that miR-338-3p down-regulation correlated with HBx expression. Moreover, siRNA knock down of HBx expression in LO2/HBx cells (Figure 7A,B) resulted in a significant increase in miR-338-3p expression and a significant reduction of CyclinD1 as compared to the negative controls (Figure 7C,D), further supporting that HBx is necessary for cyclinD1 up-regulation through miR-338-3p.

### 2.4. CyclinD1 mRNA Levels Are Up-Regulated in Human HBV-related HCC Tissues

**Figure 8:**
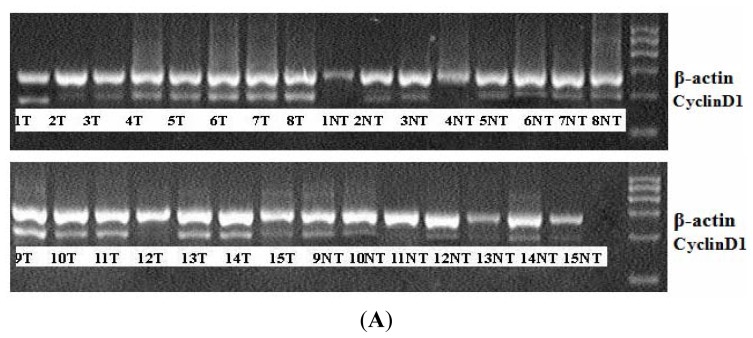

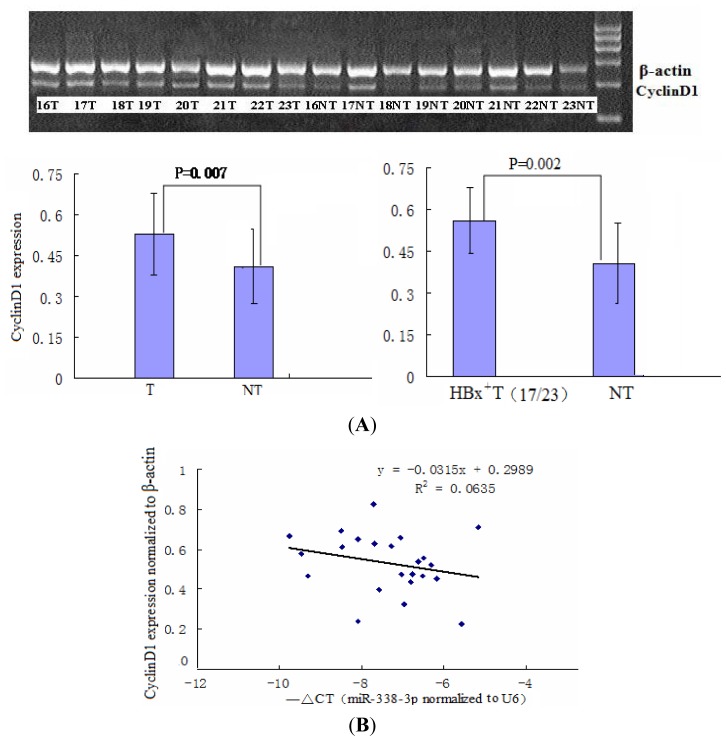
Expression of CyclinD1 mRNA levels in 23 paired tumor (T) and control non-tumor (NT) tissues from HCC patients. (**A**) CyclinD1 was up-regulated in all 23 tumor tissues (especially in the 17 HBx-positive tissues) as compared to their adjacent control non-tumor tissues as evaluated by RT-PCR; CyclinD1 expression was normalized to β-actin. (**B**) CyclinD1 mRNA expression inversely correlated with miR-338-3p expression in the 23 paired tissue samples. Correlation was statistically significant as determined by Pearson’s correlation analysis (*p* = 0.029, *r* = −0.323).

Since we show that miR-338-3p down-regulates CyclinD1 expression in our *in vitro* cell model system, we used RT-PCR to measure whether CyclinD1 mRNA expression also inversely correlated with miR-338-3p expression in HCC tissues. We first determined that CyclinD1 expression was significantly higher in tumor tissues as compared to non-tumor tissues (Figure 8A), which was especially evident in the 17 HBx-positive tumor tissues. Furthermore, although the data was highly variable due to the inherent variability in clinical samples from human tissue, a statistically significant inverse correlation was observed between miR-338-3p and CyclinD1 mRNA expression in HCC tissues (Figure 8B, *p <* 0.05). These data suggested a reciprocal regulation of the tumor suppressor miR-338-3p and its cell-cycle regulation target CyclinD1 in HBx-related HCC tissues, and indicated that miR-338-3p may play a causal role in the transformation of HBV-infected liver cells into HBx-mediated liver cancer.

### 2.5. Changes in CyclinD1 Expression Do Not Affect miR-338-3p Expression in LO2/HBx Cells

**Figure 9:**
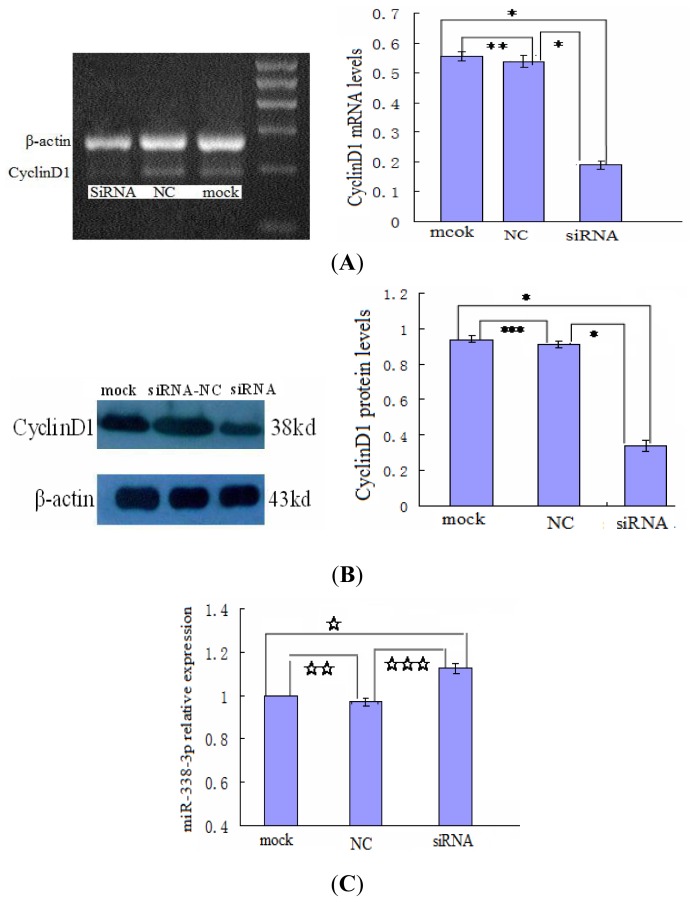
CyclinD1 does not regulate miR-338-3p expression. (**A**,**B**) CyclinD1 siRNA transfection knocked down CyclinD1 mRNA (**A**) (* *p* < 0.001, ** *p* = 0.409) and protein (**B**) (* *p* < 0.001, *** *p* = 0.289) expression in LO2/HBx cells as compared to control siRNA and mock-transfected cells. (**C**) Relative expression of miR-338-8p as measured by qRT-PCR using the 2^−ΔΔCT^ method after siRNA-induced reduction of CyclinD1 expression in LO2/HBx cells (^⋆^*p* = 0.278, ^⋆ ⋆^*p* = 0.676, ^⋆ ⋆ ⋆^*p* = 0.101). Data are shown as mean ± SD of three separate experiments.

Since the CyclinD1 protein expression inversely correlated with miR-338-3p expression, we wondered whether CyclinD1 could also regulate miR-338-3p expression, where reduced CyclinD1 would lead to increased miR-338-3p expression. We transfected CyclinD1 siRNA into LO2/HBx cells to inhibit CyclinD1 expression (Figure 9A,B) and measured miR-338-3p expression by qRT-PCR after 48 h of transfection Compared to the negative control RNA and mock transfected cells, we found that miR-338-3p expression did not significantly change after CyclinD1 knock down (Figure 9C), suggesting that CyclinD1 does not regulate miR-338-3p expression.

## 3. Discussion

The HBx protein is a tumorigenic protein that is associated with HCC development and progression. Animal model experiments have shown that transgenic mice expressing the HBx protein were susceptible to HCC [16]. Previous reports suggested that HBx triggers changes in cell behavior through its interaction with a variety of host cellular factors [17]. In this study, we showed that HBx was associated with the down-regulation of miR-338-3p expression; this microRNA normally functions to inhibit cell proliferation by targeting positions 2397–2403 in the 3′-UTR of the cell-cycle regulatory protein, CyclinD1. Our study helps to further our understanding of the miRNA-mediated mechanism underlying HBx-associated transformation of HCV-infected liver tissue to HCC.

The miR-338 gene is located on chromosome 17q25 within intron 8 of the apoptosis-associated tyrosine kinase (AATK) gene [18], which plays an essential role in promoting neurite extension in developing neurons [19]. Our unpublished data indicated that miR-338-3p, one of the two mature forms of miR-338, was down-regulated in LO2 cells that stably expressed HBx compared to LO2/pcDNA or LO2 cells, similar to the results found in another HBV-expressing cell line HepG2.2.15 cells [20]. In present study, we show that this might be clinically relevant, as miR-338-3p was found to be down-regulated in HBV-infected HCC tumor tissues tested compared to the adjacent non-tumor tissues (Figure 1A), similar to the results found in a previous study [21]. This indicates the possibility that HBV DNA could directly affect miR-338-3p expression by disrupting gene sequences upon integration into the host genome that then affect miR-338-3p specifically or miRNA expression in general. However, the effect of HBV infection on miR-338-3p expression was HBx-specific because miR-338-3p was only significantly reduced in the 17 HBx-positive tumor tissues in which the adjacent non-tumor tissues expressed no or low HBx, as determined by RT-PCR (Figure 1B-C). Although data derived from semi-quantitative data from clinical samples can be highly variable, we found that miR-338-3p expression inversely correlated with the HBx protein in the paired tumor versus non-tumor tissues determined by immunohistochemistry (Figure 2C). Based on our results, further evaluation of a larger sample size of HBV-infected HCC tumors is likely to yield even more convincing inverse correlation; additionally, some variability may derive from other regulators of HBx or miR-338-3p expression that may be present in individual patient samples that are not taken into account in this assay. Taken together, our data provide clinical evidence that HBx down-regulates miR-338-3p expression in HCC, as was observed in the LO2/HBx cell line studies.

To understand possible mechanism(s) underlying the miR-338-3p-mediated increase in cell proliferation in HBx-related HCC, we used TargetScan to identify potential miR-338-3p target genes. The loss- and gain-of-function analyses indicated that miR-338-3p prolonged the time cells spent in the G1 phase and reduced the population of cells in S phase (Figure 4A–C), indicating that miR-338-3p suppressed DNA synthesis in LO2/HBx cells, and therefore inhibited cell proliferation. Luciferase reporter assays demonstrated that miR-338-3p significantly reduced the expression of the wild-type CyclinD1 compared with the mutant CyclinD1 (Figure 6B), mainly through positions 2397–2403 of the CyclinD1 3′-UTR (Figure 6C). Furthermore, the overexpression and down-regulation of miR-338-3p reduced and enhanced CyclinD1 protein expression in LO2/HBx cells, respectively (Figure 6E), while the loss of CyclinD1 did not influence miR-338-3p expression (Figure 9D). Therefore, we identified CyclinD1 as a key target gene that regulates the G1 phase during cell proliferation [22] and suggest that CyclinD1 is a direct target of miR-338-3p.

Multiple lines of evidence in the field support this hypothesis. For example, survivin, a member of a family of apoptosis inhibitor proteins, was upregulated in many cancers, including HCC. Survivin has been mapped to chromosomal region 17q25 [23], a region shared by the miR-338 locus, and is frequently increased in advanced stages of neuroblastoma; survivin is also significantly associated with poor clinical outcome [24]. This indicates that miR-338-3p is located in a fragile locus associated with tumor formation and may play an important role in tumor development. miR-338-3p was reported to target several important cellular factors, such as COXIV [25] and SMO [26], the latter of which seems to trigger a series of intracellular events and induces the expression of numerous target genes that regulate proliferation, differentiation, and extracellular matrix interactions [27]. In this study, we experimentally validated another direct cellular target of miR-338-3p, CyclinD1. CyclinD1 is a member of the G1 phase family of cyclins that are modulated by extra- or intracellular mitotic signals and promotes the progression of the cell cycle from the G1 to S phase through the activation of cyclin-dependent kinases (CDK), namely CDK4 and CDK6 [28]. CyclinD1 overexpression is an important factor in tumor invasiveness and metastasis and is more sensitive to cell cycle regulation in comparison to other cyclins [29]. Likewise, CyclinD1 inhibition by RNAi inhibits matrix metalloproteinase (MMP) expression and KM20 cell proliferation by causing cell cycle arrest [30]. Therefore, CyclinD1 is also considered an “oncogene” [28].

Previous studies suggested that HBx can induce liver cells to enter the cell cycle, or progress through the cell cycle more rapidly by stimulating Src kinases and cyclin gene expression [31] or increasing the rate and level of activation of the cyclin-dependent kinases and RAS [32]. Moreover, HBx increases the the levels of CyclinD1 in cultured primary rat hepatocytes [33] or in Chang liver cells through extracellular mitotic signals [34] or up-regulation of NF-κB2(p52)/BCL-3 in the nucleus [35]. As cyclinD1 is a member of cyclins, upon these previous data, we can infer that the effect of HBx on cell cycle may be related with the up-regulation of CyclinD1, which supports our results. Our findings indicated that CyclinD1 was up-regulated in HBx-positive HCC tumor tissues compared to paired control non-tumor tissues, and there was an inverse correlation between CyclinD1 expression and miR-338-3p expression in HCC tissues, which was consistent with our *in vitro* results. The down-regulation of miR-338-3p in HBx-positive tissues, resulting in an up-regulation of CyclinD1, indicates that miR-338-3p may act normally as a tumor suppressor in HCC development. Similarly, several recent studies have identified miR-503 [36], miR-193b [37], miR-378 [38], miR-520b [39] and miRNA-365 [40] as suppressors of CyclinD1. The down-regulation of these miRNAs enhanced CyclinD1 gene expression and led to tumor cell proliferation, suggesting that CyclinD1 may be tightly regulated by several different miRNAs, including miR-338-3p. miR-338-3p may therefore function as a tumor suppressor in HBV-related HCC development.

In summary, this report highlighted a novel carcinogenic role for HBx in HCC development through the down-regulation of miR-338-3p expression. The nt 2397–2403 position of the CyclinD1 3′-UTR may be the major target through which miR-338-3p inhibits cell proliferation. The deregulation of miR-338-3p expression by HBx may represent a potential novel pathway through which HBx acts to deregulate cell proliferation, leading to hepatocarcinogenesis; thus, these findings may have potentially relevant therapeutic implications.

## 4. Experimental Section

### 4.1. Patient Samples

The 23 paired HBV-related HCC and the adjacent liver tissue samples used in this study were obtained from patients who underwent radical resection at Xiangya Hospital (Central South University, Changsha, China). The samples were frozen immediately after being surgically resected from the patients and stored in liquid nitrogen. The identities of both the tumor and noncancerous samples were confirmed histologically. HBV infection was diagnosed when HBV surface antigen (HBsAg) was detected by ELISA in serum isolated from peripheral blood. All HCC tumors originated from the background diagnosis of chronic hepatitis or cirrhosis. Informed consent was obtained from each patient. The study was performed according to the guidelines of the institutional review board of the Liver Cancer Institute.

### 4.2. Immunohistochemistry

All paraffin-embedded liver tissues were stained with hematoxylin and eosin (H & E) for the analysis of morphological changes. Immunohistochemical staining was performed to detect the expression of HBx in HCC tumor tissues and in patient-matched non-cancerous tissues. The primary antibody against HBx was obtained from Abcam Biosciences and used at a 1:10 dilution. HBx protein expression level was scored by semi-quantitative analysis of the staining according to the following criteria [41]. 5 fields were randomly selected for each sample, and at least 100 cells were counted for each field. Scoring was first measured by the percentage of HBx-positive cells within the sample: less than 5% scored 0; 5–24% scored 1; 25–49% scored 2; 50–74% scored 3; more than 74% scored 4. In addition, HBx protein expression level within each positive cell was scored by staining intensity: no coloring 0; faint yellow 1; clay bank 2; tawny 3. The intensity color and scores were combined into one final score based on the two criteria: negative (−) scored 0; weakly positive (+) scored 1~4; moderately positive (++) scored 5~8; strongly positive (+++) scored 9~12.

### 4.3. Cell Culture and the Establishment of Stably Transfected Cell Lines

Our group previously generated the pcDNA3.0 and pcDNA/HBx plasmids (containing restructured HBx genetic fragments originating from the liver cell line HepG2.215), and cell lines stably expressing these plasmids were established as previously described [42]. Cells were cultured in RPMI-1640 medium (GIBCO, Erie, NY, USA) containing 10% fetal bovine serum (FBS, GIBCO, Erie, NY, USA) and 100 units/mL penicillin plus 100 μg/mL streptomycin at 37 °C with 5% CO_2_.

The hepatocyte cell line LO2 (obtained from the Department of Cell Biology, Chinese Academy of Science, Shanghai Institute, Shanghai, China) was transfected with Lipofectamine^TM^2000 (Invitrogen, Carlsbad, CA, USA) according to the manufacturer’s instructions and subsequently selected with G418 (Geneticin, GIBCO, Erie, NY, USA). An empty pcDNA3.0 vector plasmid was used as a control. The stable transfection of pcDNA/HBx (termed LO2/HBx) or the empty vector (termed LO2/pcDNA3.0) was confirmed by RT-PCR and western blotting.

### 4.4. Cell Transfection

Transfections were performed with a Lipofectamine2000 kit according to the manufacturer’s instructions. A double-stranded miR-338-3p mimic, single-stranded miR-338-3p inhibitor, or a related negative control RNA (GeneCopoeia, Rockville, MD, USA) was introduced into LO2/HBx cells at a final concentration of 50 nM. Transfected cells were harvested 48 h later.

### 4.5. Inhibition of HBx/CyclinD1 Expression

Small interfering RNA (siRNA) sequences specifically targeting HBx were synthesized by Ribobio (Guangzhou, China), while the siRNA sequences specifically targeting CyclinD1 were synthesized by GenePharma (Shanghai, China). Approximately 50 nM HBx/CyclinD1 siRNA or control siRNA was transfected into LO2/HBx cells by Lipofectamine as described above.

### 4.6. Quantitative Real-Time PCR (qRT-PCR) Analysis

CyclinD1 expression in LO2/HBx cells transfected with miR-338-3p mimics or miR-338-3p inhibitor was measured with SYBR RT-qPCR. miR-338-3p expression in 23 paired HCC/adjacent tissues was measured with SYBR RT-qPCR. Total RNAs were extracted with Trizol (Invitrogen) according to the manufacturer’s instructions.

miR-388-3p cDNA was synthesised from 2 μg of total RNA with an All-in-one^TM^ miRNA First-Strand cDNA Synthesis (GeneCopoeia) Kit using the supplied poly-A primer. Real-time PCR was performed in a 20 μL reaction mix including 2 μL of 5× diluted reverse transcription product, 2 μL miRNA specific primer, 10 μL SYBR 2× All-in-one qPCR Mix, 0.4 μL 50× ROX reference dye and 3.6 μL double distilled water. The cycling conditions for amplification on the 7500 Real-Time PCR System (Applied Biosystems, Foster City, CA, USA) were 95 °C for 10 min, followed by 40 cycles of 95 °C for 10 s, 60 °C for 20 s, and 72 °C for 32 s. The data were normalized against the U6 snRNA.

CyclinD1 expression was analyzed with THUNDERBIRD SYBR qPCR Mix (ToYoBo, Osaka, Japan). cDNA was synthesized with the RevertAid^TM^ First Strand cDNA Synthesis Kit (MBI Fermentas, Ontario, Canada) in a total volume of 20 μL. The primer sequences used were as follows: for CyclinD1, 5′-AGGAACAGAAGTGCGAGGAGG-3′ (forward) and 5′-GGATGGAGTTGTCG GTGTAGATG-3′ (reverse); for GAPDH, 5′-CGGATTTGGTCGTATTGGGC-3′ (forward) and 5′-CCTGGAAGATGGTGATGGGATT-3′ (reverse). qRT-PCR was performed on an Applied Biosystems 7500 RT-PCR System. The cycling conditions for amplification were 95 °C for 1 min, 40 cycles of 95 °C for 15 s, and 58 °C for 35 s. The data were normalized against GAPDH.

Each sample was analyzed in triplicate. Fluorescence signal was measured at each extension step. The relative expression was determined with the 2^−ΔΔCT^ method, where the normalized CT (ΔCT) was calculated by subtracting the CT of a control gene (U6 snRNA for miR-338-3p and GAPDH for CyclinD1) from the CT of the gene of interest.

### 4.7. RT-PCR and Western Blot Analysis

RT-PCR was used to measure the HBx and CyclinD1 expression in 23 paired HCC/adjacent tissues and to measure the CyclinD1 expression in LO2/HBx cells before and after knockdown with siRNA or the negative control. As a control for contaminating DNA in the RT-PCR data, no bands were observed when RNA samples were tested before undergoing RT (data not shown).cDNA was synthesized with the RevertAid^TM^ First Strand cDNA Synthesis Kit (MBI Fermentas). The primer sequences used were as follows: for the 462 bp HBx product, 5′-AAGGTACCATGGCTGCTAGGCTGTGCT-3′ (forward) and 5′-CTGGGCCCTTAGGCAGAGGTGAAAAAGTTG-3′ (reverse); for the 192 bp CyclinD1 product, 5′-AGGAACAGAAGTGCGAGGAGG-3′ (forward) and 5′-GGATGGAGTTGTCGGTGTA GATG-3′ (reverse); for the 242 bp β-actin product, 5′-CTCCATCCTGGCCTCGCTGT-3′ (forward) and 5′-GCTGTCACCTTCACCGTTCC-3′ (reverse). Amplification conditions were 94 °C for 3 min, followed by 35 cycles of 94 °C for 30 s, 58 °C for 30 s, and 72 °C for 1 min, followed by an elongation cycle of 72 °C for 5 min. The resulting PCR products were analyzed by electrophoresis on 1.5% TBE agarose gel using β-actin as an internal control.

CyclinD1 protein expression was measured by western blot. Total protein was extracted from transfected cells using the RIPA lysis buffer (Beyotime, Shanghai, China) according to the manufacturer’s instructions. For western blot analysis, equal amounts of protein for each sample were separated by 10% SDS-PAGE and electroblotted onto PVDF membranes (Millipore, Billerica, MA, USA). Blots were blotted with 5% skim milk, followed by incubation with antibodies specific for mouse anti-CyclinD1 at a 1:100 dilution (Santa Cruz, Santa Cruz City, CA, USA), and mouse anti-β-actin at a 1:3000 dilution (Abcam). Blots were then incubated with goat anti-mouse secondary antibody conjugated to horseradish peroxidase at a 1:5000 dilution (Jackson Immunoresearch, Philadelphia, PA, USA) and visualized by enhanced chemiluminescence (ECL) (Amersham Biosciences, Piscataway, NJ, USA).

### 4.8. Luciferase Reporter Assay

The full-length 3′-UTR of CyclinD1 (Genbank accession No. NM_053056) containing the putative miR-338-3p binding sequences (907–913 nt, 2397–2403 nt) was amplified using cDNA from LO2/HBx cells cloned into the XhoI and NotI restriction sites of pmirGLO Vector (Promega, Madison, WI, USA). The recombinant reporter vector carrying the wild-type CyclinD1 3′-UTR was termed pCyclinD1-3′-UTR-WT. Site-directed mutagenesis of the miR-338-3p target sites in the CyclinD1-3′-UTR was carried out by PCR mutagenesis where both of the two putative miR-338-3p binding sites were mutated with the plasmid constructed as above (termed pCyclinD1-3′-UTR-Mut). Each of putative miR-338-3p binding sequences were also separately mutated, and the constructed plasmids were termed pCyclinD1-3′UTR-Mut1 (mutated 907–913 nt) and pCyclinD1-3′-UTR-Mut2 (mutated 2397–2403 nt). We verified that the mutant sites did not bind to known human miRNAs *in silico* using miRBase (Release 12.0; Sanger Institute: Cambridge, UK, 2008). Both constructs were verified by sequencing.

The primers used for cloning were as follows: **Wt**-Forward: 5′-CCGCTCGAGTCCTATTTTTGTAGTGACCTGTTTATG-3′ and **Wt**-Reverse: 5′-GAATGCGGCCGCGCTACGCCCCCGATCAGATGAAG-3′; **Mutant**-Forward: 5′-CCGCTCGAGTCCTATTTTTGTAGTGACCTGTTTATGAG***TTCCAGA***TTTTCTACCCAACGGCCC-3′ and **Mutant**-Reverse: 5′-GAATGCGGCCGCGCTACGCCCCCGATCAGATGAAGTGC***TCTGGAA***CACAGGCGCAGGGAAGAGAAG-3′; **Mutant1**-Forward: 5′-CCGCTCGAGTCCTATTTTTGTAGTGACCTGTTTATGAG***TTCCAGA***TTTTCTACCCAACGGCCC-3′ and **Mutant1**-Reverse: 5′-GAATGCGGCCGCGCTACGCCCCCGATCAGATGAAG-3′; **Mutant2**-Forward:5′-CCGCTCGAGTCCTATTTTTGTAGTGACCTGTTTATG-3′ and **Mutant2**-Reverse: 5′-GAATGCGGCCGCGCTACGCCCCCGATCAGATGAAGTGC***TCTGGAA***CACAGGCGCAGGGAAGAGAAG-3′.

For reporter assays, pCyclinD1-3′-UTR-WT, pCyclinD1-3′-UTR-Mut, pCyclinD1-3′-UTR-Mut1, and pCyclinD1-3′-UTR-Mut2 reporter constructs (100 ng/mL) were co-transfected into LO2/HBx cells in 96-well plates with 50 nM miRNA mimics or a negative control using Lipofectamine2000. Reporter assays were performed 48 h post-transfection using the Dual-Luciferase Reporter Assay system (Promega, Madison, WI, USA) to quantify luminescent signal using a luminometer (Veritas^TM^ microplate fluorescence reader; YuanPingHao Bio, Beijing, China). Each value from the Renilla luciferase assay was normalized to the Firefly luciferase value from the co-transfected pmirGLO vector (Promega) to correct the differences in both transfection and harvest efficiencies. Transfections were done in duplicate and repeated at least three times in independent experiments.

### 4.9. EdU Assay

Cell proliferation was measured by 5-ethynyl-2′-deoxyuridine (EdU) assay using a EdU assay kit (Ribobio, Guangzhou, China) according to the manufacturer’s instructions. Briefly, LO2/HBx cells at 5 × 10^3^ cells per well were cultured in triplicate in 96-well plates and transfected with 50 nM of miR-338-3p mimics, miR-338-3p inhibitor, or their relative control RNA, respectively, for 48 h. The cells were then exposed to 50 μM of EdU for additional 4 h at 37 °C. The cells were fixed with 4% formaldehyde for 15 min at room temperature and treated with 0.5% Triton X-100 for 20 min at room temperature for permeabilization. After washing 3× with PBS, each well containing cells was treated with 100 μL of 1× Apollo^®^ reaction cocktail for 30 min. Subsequently, the cellular DNA contents of each well were stained with 100 μL of Hoechst 33342 (5 μg/mL) for 30 min and visualized under a fluorescent microscope (Olympus, Tokyo, Japan).

### 4.10. Cell Cycle Assay

Cell cycle analysis was determined by flow cytometry (BD, Franklin Lakes, NJ, USA). Briefly, LO2/HBx cells at 1 × 10^6^ cells per well were cultured in 6-well plates and transfected with 50 nM of miR-338-3p mimics, miR-338-3p inhibitor, or their relative control RNA, respectively, for 48 h. The cells were harvested and fixed in 70% ice-cold ethanol for 24 h, followed by staining with propidium iodide (PI). The different phases of the cell cycle were analyzed using a FACS Calibur instrument.

### 4.11. Statistical Analysis

The relationship between miR-338-3p mRNA and HBx protein expression as well as between miR-338-3p and CyclinD1 mRNA expression were analyzed by Pearson’s correlation. The other data were analyzed by unpaired two-tailed *t*-test. All data was expressed as mean ± standard deviation from at least three independent experiments. All *p* values were obtained with SPSS 16.0 software package (*SPSS*, version 16.0; SPSS Inc: Chicago, IL, USA, 2007), and a *p* < 0.05 was considered statistically significant.

## 5. Conclusions

miR-338-3p Could be down-regulated by HBx and inhibits cell proliferation majorly through targeting nt 2397-2403 position of CyclinD1 3′UTR. The deregulation of miR-338-3p expression by HBx may represent a potential novel pathway through which HBx acts to deregulate cell proliferation, leading to hepatocarcinogenesis and may facilitate therapeutic development against HCC.

## Supplementary Information
