# Supplementary material for: miR-338-3p Is Down-Regulated by Hepatitis B Virus X and Inhibits Cell Proliferation by Targeting the 3′-UTR Region of CyclinD1
Source: Int J Mol Sci. 2012 Jul 9;13(7):8514–39. doi: 10.3390/ijms13078514 (PMC3430248; doi:10.3390/ijms13078514)
Supplement: Supplementary file 1 [file ijms-13-08514-s001.pdf]

miR-338-3p Is Down-Regulated by Hepatitis B Virus X and Inhibits Cell Proliferation by Targeting the 3′-UTR Region of CyclinD1

Supplementary Information

Table S1. Clinicopathological features and miR-338-3p expression of 23 patients with HCC.

Clinical information	No. of cases	miR-338-3p (−ΔCT value)	*p* value
Age (years)			>0.05
>40	10	−6.717 ± 0.201	-
<40	13	−6.501 ± 0.489	-
Gender			>0.05
male	18	−6.587 ± 0.727	-
female	5	−6.693 ± 0.193	-
Tumor differentiated			<0.05
well	7	−5.855 ± 0.179	-
moderately	12	−6.518 ± 0.238	-
poorly	4	−6.973 ± 0.214	-
The presence of liver cirrhosis of non-tumorous tissue			>0.05
Yes	15	−6.732 ± 0.291	-
No	8	−6.590 ± 0.211	-

Note: Clinicopathological characteristics and miR-338-3p expression of the patients. As shown in the table, miR-338-3p expression was reversely related with tumour grade. There was a tendency for miR-338-3p expression to decrease with the progression of tumor grade (poorly differentiated < moderately differentiated < well differentiated). However, there was no significant difference between miR-338-3p expression and other clinicopathological parameters in HCC, including state of age, gender, and liver cirrhosis in surrounded tumor tissue.

Table S2. The percentage of cells at G1/S stage of the cell cycle.

Cells	Groups	G1	S
LO2/HBx	mimics-NC	70.59 ± 1.07	22.01 ± 1.27
	mimics	83.82 ± 1.05	10.57 ± 0.87
	inhibitor-NC	71.1 ± 1.09	20.41 ± 1.39
	inhibitor	59.55 ± 1.1	27.0 ± 0.33
LO2/pcDNA3.0	mimics-NC	76.10 ± 1.68	17.58 ± 1.71
	mimics	83.01 ± 1.1	10.87 ± 1.31
	inhibitor-NC	77.20 ± 1.57	15.86 ± 0.86
	inhibitor	70.12 ± 1.73	19.97 ± 1.31

© 2012 by the authors; licensee MDPI, Basel, Switzerland. This article is an open access article distributed under the terms and conditions of the Creative Commons Attribution license (http://creativecommons.org/licenses/by/3.0/).
